# Blood B Cell and Regulatory Subset Content in Multiple Sclerosis Patients

**DOI:** 10.4172/2376-0389.1000139

**Published:** 2015-05-04

**Authors:** Jakob Habib, Jiusheng Deng, Neil Lava, William Tyor, Jacques Galipeau

**Affiliations:** 1 Department of Hematology and Medical Oncology, Winship Cancer Institute, Emory University School of Medicine, 1365 Clifton Rd NE, Atlanta, Georgia 30322, USA; 2 Department of Neurology, Emory MS Center, 1365 Clifton Rd NE, Atlanta, Georgia, 30322, USA; 3 Department of Neurology (GEC), Atlanta VA Medical Center, 1670 Clairmont Road, Decatur, GA 30033

**Keywords:** Multiple sclerosis, Flow cytometry, B cells, Regulatory B cells, IL-10, Rituximab

## Abstract

**Objective:**

B cell targeted therapies have been effective in slowing multiple sclerosis (MS) disease progression suggesting a direct causal link for this lymphoid subset. A small subset of B cells with regulative properties (Bregs) exists in peripheral blood, and induction of Bregs ameliorates experimental autoimmune encephalomyelitis (EAE), the murine model for MS. Therefore the frequency of B cell subsets and regulatory B cells in particular in peripheral blood of MS patients is of interest.

**Methods:**

The phenotype and frequency of B cell subsets in peripheral blood from 32 MS patients and 34 healthy controls (HC) were examined using flow cytometry.

**Results:**

We found that there is an increase in CD19^+^ cell number in MS 1347 ± 159 cells/μL, (average ± SEM) compared to HC, 935 ± 129 cells/μL and no apparent deficiency in B-cells with a regulatory phenotype. In addition, we observed a loss of correlation between CD19^+^ B cells and total lymphocyte count in MS.

**Conclusion:**

These findings suggest altered blood B-cell homeostasis in MS patients.

## Introduction

Multiple Sclerosis (MS) is an autoimmune disease caused by inflammatory damage to the myelin and axons in the central nervous system (CNS). Both environmental and genetic factors are important in disease onset and progression [1-4]. Until recently, MS has traditionally been described largely as a T cell mediated disease where activated T cells target and damage the myelin sheath around CNS cells. However, a recent study discovered that depletion of peripheral B cells with the anti-CD20^+^ monoclonal antibody rituximab (Rituxan^®^, Genentech and Biogen Idec) leads to a rapid decline of disease activity in MS [5]. The role of B cells in MS pathogenesis has thenceforth been of greater focus. B cells perform several roles in the immune system, one of which is to secrete antigens that activate T cells. One study revealed that B cells of MS patients respond more robustly to activation, such as from a secondary infection, leading to higher levels of T cell activation and therefore increased CNS damage [6]. Another study induced experimental autoimmune encephalomyelitis (EAE), the murine model for MS, in either a B cell dependent or independent manner [7]. The B cell dependent-induced EAE caused B cell activation and differentiation into antigen presenting cells (APCs), which subsequently activated T cells. When the mice were anti-CD20 depleted, EAE progression halted and in some cases reversed. In contrast, the B cell independent-induced EAE was exacerbated when the mice were anti-CD20 depleted. This study demonstrated the dual-functional role that B cells provide as both pathogenic and regulative. Clinically, the presence of high levels of immunoglobulin G (IgG) in the cerebral spinal fluid (CSF), detected as oligoclonal bands, is a main factor that physicians consider in the diagnostic workup of MS. It is believed that these oligoclonal bands are produced by clonally expanded plasma cells derived from B cells present in the borders of the blood brain barrier [8]. These studies demonstrate that B cells play a role in MS, but their exact function in the pathogenesis of the disease has yet to be elucidated.

The disease modifying drugs (DMDs) currently available to treat MS affect relapsing remitting multiple sclerosis disease progression. Interferons mediate the immune system by altering cytokine production, inhibiting T-cell activation, and decreasing the expression of MHC Class II molecules [9]. Glatiramer acetate mediates the disease progress of RRMS patients by shifting Th1 cells to Th2 cells. These Th2 cells secrete anti-inflammatory cytokines such as IL-5 and IL-13 [10]. Fingolimod is different because it readily penetrates the blood-brain barrier and acts on both the immune system and CNS by inhibiting recirculation of lymphocytes from lymph nodes [11]. These treatments function to decrease the frequency and severity of relapses, but can cause unpleasant side effects and potential risks in pregnancy [12,13].

B cells mature in the bone marrow, can circulate through the blood and lymphatic system, and are identified by their expression of CD19. Naïve B cells express Immunoglobulin D (IgD) on their surface and can differentiate into memory B cells when activated by either T cell dependent or independent antigens. These memory B cells are long-lived, specific to the initially encountered antigen, and identified by their expression of CD27 [14]. While the majority of B cells stimulate the immune system and contribute to antigen clearance and inflammation, some B cells Rpress immune functions. These regulatory B cells (Bregs) are a small subset of B cells with CD19^+^CD5^+^CD1d^+^ surface markers in mice [15] and it has been shown that treatment with autologous Bregs in mice ameliorates EAE [16]. Bregs produce a diversity of immunomodulating mediators, with the most predominant and well-established factor being interleukin 10 (IL-10). IL-10 broadly Rpresses the immune system by modulating the function of innate and adaptive responses. It acts on APCs to inhibit production of cytokines by Th1 (T helper 1) cells, and this reduction of Th1 response is a key mediator in EAE recovery [17,18]. Also, one study found that levels of IL-10 are lower in untreated MS patients compared to patients receiving anti-inflammatory drugs [19]. Therefore, the biology of B-cells and Bregs in particular are of mechanistic interest in MS patients. Consequently, we examined the number of naïve, memory, and regulatory B cells in MS patients relative to normal as to inform whether there are any distinguishing distributions which may inform a plausible association.

## Methods

Thirty-two MS whole blood samples were collected from consenting adults at the Emory MS clinic. Thirty-four healthy controls (HC) blood samples were examined as well. Both MS and HC subjects had ages>18. We included three types of MS in this study: relapsing-remitting (RRMS), primary progressive (PPMS), and secondary progressive (SPMS) [20]. The sex, disease state, and DMDs of MS patients are listed in Table 1. An absolute lymphocyte count was obtained for 11 MS and 15 HC within a week of sample collection.

**Figure 1:**
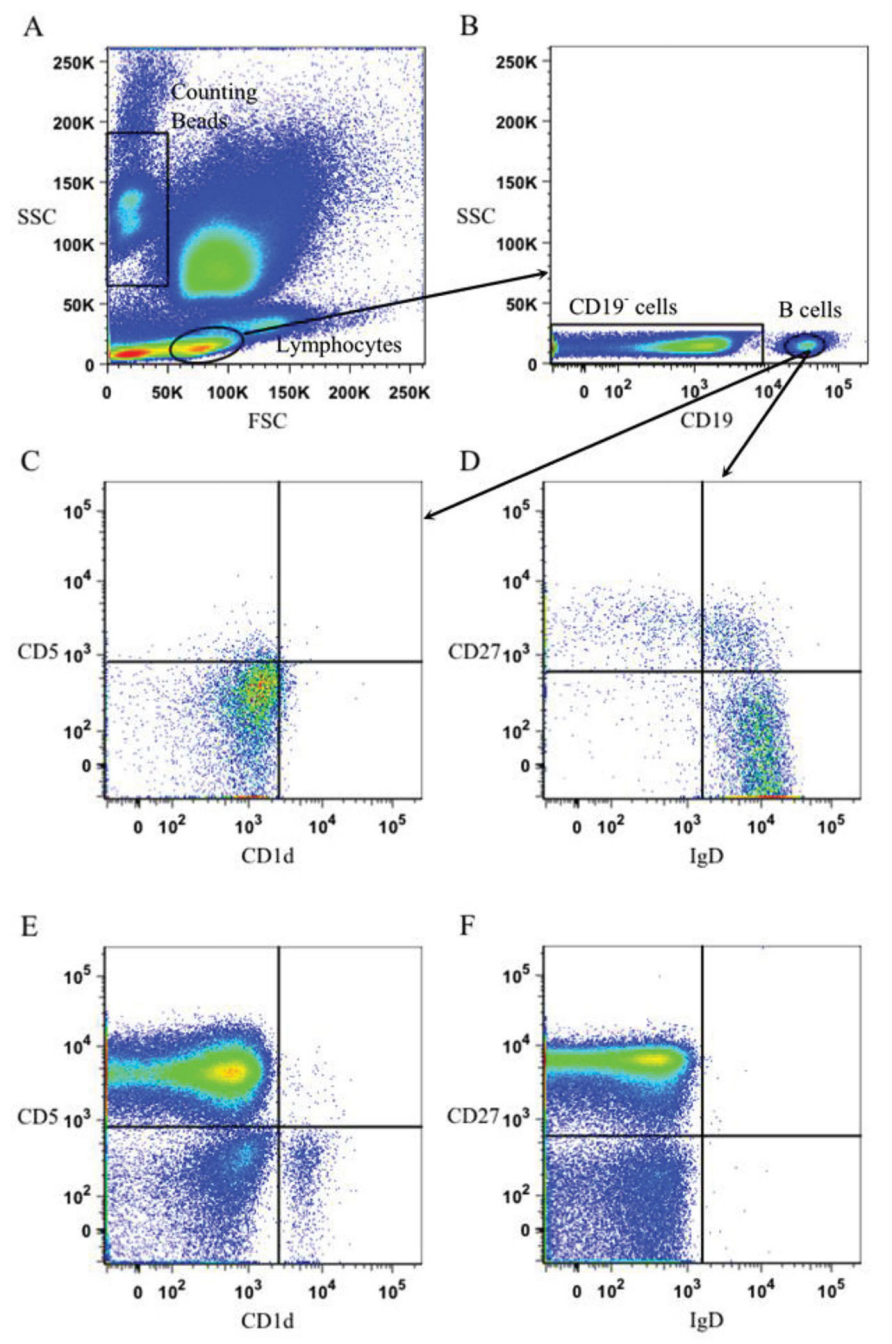
Whole blood flow cytometric analysis. **A:** Forward scatter (FSC) measures cell size and side scatter (SSC) measures cell granularity. Counting Beads and Lymphocyte gates are depicted. **B:** Lymphocyte gating revealed a CD19^+^ (B cell) population and a CD19^−^ population. **C:** Bregs are defined as CD19^+^CD5^+^CD1d^+.^ **D:** Memory B cells are defined as CD19^+^CD27^+^IgD^−^ and naïve B cells are defined as CD19^+^CD27^−^IgD^+^. **E:** The CD19^−^ subset from Figure 1B revealed clear CD5 and CD1d populations which were used to establish gating in Figure 1C. **F:** The CD19^−^ subset also revealed clear CD27 and IgD populations which were used to determine gating in Figure 1D.

Whole blood samples (3-5 mL) were collected in EDTA Vacutainer tubes (BD). Peripheral whole blood samples (100 μL) were stained with 5μL anti-CD19-PE-Cy7, anti-CD1d-PE, anti-CD5-FITC, and anti-CD27-APC, and 2 μL anti-IgD-Per CP for 20 minutes at room temperature. Following staining, 500μL of red blood cell (RBC) lysis buffer containing tris buffered ammonium chloride was added and the sample was incubated for 10 minutes at room temperature. Phosphate-Buffered Saline (500 μl) was then added, followed by 50 μL AccuCheck Counting Beads (Invitrogen) to quantify the B cell subsets. The sample in its entirety was processed by flow cytometry on a BD FACS Canto II machine. Data was collected on BD FACS Diva software and analyzed with Flow Jo. Distinct B cell subsets were defined utilizing the gating strategy indicated in Figure 1. B cells are CD19^+^, Bregs are CD19^+^CD5^+^CD1d^+^, memory B cells are CD19^+^CD27^+^IgD^−^, naïve B cells are CD19^+^CD27^−^IgD^+^, and naïve Bregs are CD19^+^CD27^−^IgD^+^CD5^+^CD1d^+^. A student’s two-tailed t-test assuming unequal variance was used to determine the significance between MS and HC. T tests, linear regression lines, and two-way ANOVAs were calculated using Prism GraphPad.

## Results

**Figure 2:**
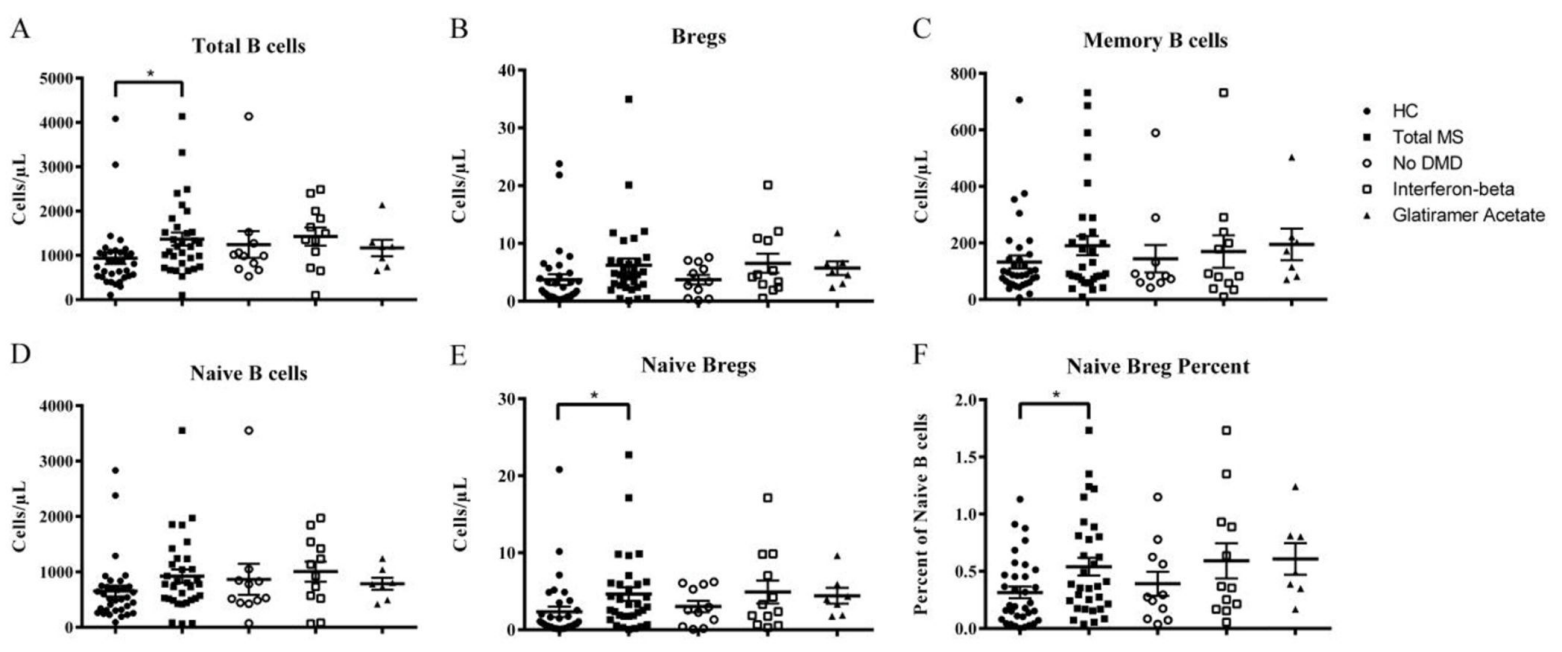
Analysis of whole blood samples for HC (n=34), total MS (n=32), no DMD (n=11), interferon-beta (n=12), and glatiramer acetate (n=7). Data for fingolimod was not included due to low sample size (n=2). **A:** Total number of CD19^+^ B cells. **B:** Number of CD19^+^CD5^+^CD1d^+^ Bregs. **C:** Number of CD19^+^CD27^+^IgD^−^ memory B cells. **D:** Number of CD19^+^CD27^−^IgD^+^ naïve B cells. **E:** Number of CD19^+^CD27^−^IgD^+^CD5^+^CD1d^+^ naïve Bregs. **F:** Percent of naïve B cells that are CD5^+^CD1d^+^. Error bars represent mean ± SEM. Significant difference is indicated: * p < 0.05.

There was a significant increase in the number of total B cells in MS, n=32, 1369 ± 147 cells/μL (mean ± SEM) compared to HC, n=34, 935 ± 129 cells/μL (p=0.029, Figure 2A). We did not observe a difference in the number of Bregs, memory B cells, or naïve B cells between MS and HC (Figure 2B-2D). However, there was an increase (p=0.042) in the number of CD19^+^CD27^−^IgD^+^CD5^+^CD1d^+^ naïve Bregs in MS, 4.6 ± 0.9 cells/μL compared to HC, 2.3 ± 0.7 cells/μL (Figure 2E). There was also a greater percentage of naïve Bregs in MS, 0.54 ± 0.08% versus HC, 0.31 ± 0.05% (p=0.016, Figure 2F). We did not observe any significant difference between the DMD subsets relative to MS subjects as a whole (Figure 2A-2F).

**Figure 3:**
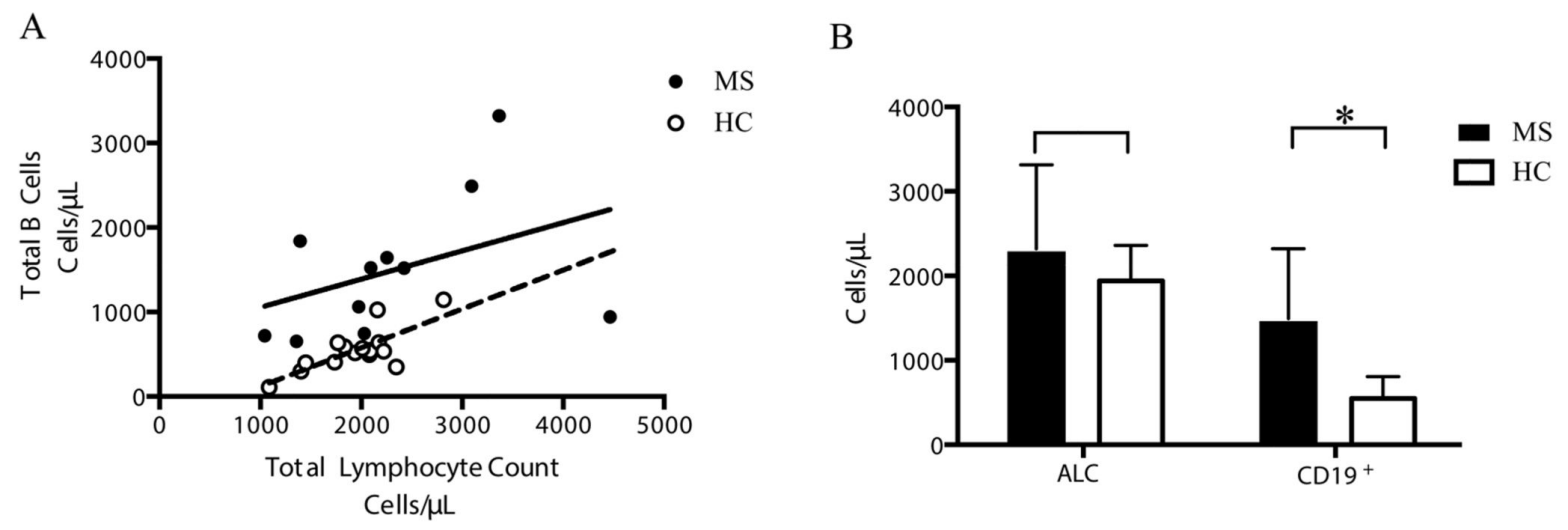
Analysis of 11 MS and 15 HC samples for which an absolute lymphocyte count (ALC) was obtained. **A:** Absolute lymphocyte count versus total number of CD19^+^ supplementary files B cells. Linear regression lines were calculated for MS (p=0.22) and HC (p<0.01). **B:** Two-way ANOVA of absolute lymphocyte count and CD19^+^ B cells. Significant difference is indicated: ^*^ p < 0.05.

Analysis of the absolute lymphocyte count (ALC) for 11 MS and 15 HC samples revealed a difference in the correlation between total lymphocyte count and CD19^+^ B cells (Figure 3A). In HC a stronger correlation exists, R^2^=0.56 (p<0.01), but in MS there is a weaker correlation, R^2^=0.16 (p=0.22), suggesting that the disease is associated with a disruption in normal B cell homeostasis. An ANOVA performed on these samples revealed that while MS has more CD19^+^ cells, 1495 ± 827 cells/μL (mean ± SD) than HC, 549 ± 260 (p<0.01), there is no significant difference between the total number of lymphocytes (Figure 3B).

## Discussion

B cell targeted therapies have become an important focus in MS research, but the precise role of B cells in MS is not fully understood. We investigated the phenotype of peripheral B cells in MS patients with varying disease types and on different DMDs. Our findings demonstrate that MS is not associated with a significant deficiency in CD5^+^CD1d^+^ Bregs, (Figure 2B). However, we found an increase in total CD19^+^ B cell number (Figure 2A) and CD19^+^CD27^−^IgD^+^CD5^+^CD1d^+^ naïve Breg number (Figure 2E) in MS patients. The percentage of CD19^+^CD27^−^IgD^+^ naïve B cells with a CD5^+^CD1d^+^ Breg phenotype increased in MS as well (Figure 2F), demonstrating that this increase in naïve Breg number is not directly related to the increase in total MS B cells. It has been proposed that IL-10 derived from naïve B cells functions to prevent autoimmune inflammatory responses, where as IL-10 derived from memory B cells functions to resolve active disease exacerbation [21]. Another study found that IL-10 is produced primarily by naïve B cells, whereas memory B cells produce proinflammatory proteins such as tumor necrosis factor alpha [22]. In addition, RRMS patients undergoing a relapse have a diminished proportion of naïve Bregs [23]. Though we do not have information on the disease activity of each individual at the time of sample collection, it would be interesting to observe if there is a correlation between B cell subsets and relapses. It is possible that remission is facilitated by an increase in naïve Bregs which function to prevent against the inflammatory response of MS. Indeed, a study utilizing induced Bregs derived from naïve B cells ameliorated EAE, and this strategy could potentially be used to produce autologous induced Bregs for personalized immunotherapy to treat MS [16].

An absolute lymphocyte count was available for patients who received a CBC within a week of sample collection. By plotting the absolute lymphocyte count against the number of CD19^+^ B cells in Figure 3 we expect a linear correlation. Instead we find that MS has a reduced correlation, indicating dissociation between total lymphoid content and CD19^+^ B cell count which suggests altered homeostasis regulation B cell content (Figure 3A). This aberrance does not directly imply that B cells are the cause of MS progression as they might be a consequence of the disease, and we only observe an association between MS and B cell number. However, if this increase in B cells is not a byproduct of MS but rather a contributing factor to the disease, it is clear that B cell depletion therapies such as rituximab would be beneficial to MS patients [5]. These observations suggest that there is possibly a distinct B cell homeostatic profile in multiple sclerosis. Interestingly, we did not observe an absolute decrease in the number of blood Bregs. This leads us to propose that Breg deficiency is not apparent in MS as a possible lymphoid defect and that there may be a disease relationship between B cell biology and MS which may explain, in part, beneficial B cell depletion strategies.

There has been extensive debate about the identification of Bregs and the role of IL-10 in autoimmune diseases. Our study defined Bregs as CD19^+^CD5^+^CD1d^+^ based on the phenotype defined in mice [15]. A more definitive method of identifying peripheral Bregs would be to measure intracellular levels of IL-10 following stimulation with PMA and ionomycin [24,25]. The CD5^+^CD1d^+^ Bregs identified in our study may be a small subset of a larger population of IL-10 producing B cells, or B10 cells, which as a whole are only identifiable by their capacity to express IL-10 [26]. While we did not observe a difference in the CD19^+^CD5^+^CD1d^+^ Breg population between MS and HC, it is possible that the overall B10 population might be distinct. The exact contribution that IL-10 provides in other autoimmune diseases is also controversial. IL-10 production was found to be higher in rheumatoid arthritis (RA), primary Sjogren’s syndrome (SS), and systemic lupus erythematosus (SLE), pointing to B cell hyperactivity as the cause of these autoimmune diseases [27]. However, in SLE patients a subset of Bregs defined as CD19^+^CD24^hi^CD38^hi^ produced less IL-10 and lacked Rpressive capacity when compared to healthy controls [28]. In addition, recombinant IL-10 has been proposed as a therapeutic treatment for RA due to its efficacy in the mouse model collagen-induced arthritis [29]. In EAE, Bregs inhibit disease initiation whereas generic B cells promote disease progression, as demonstrated in a study utilizing CD20 antibody-mediated depletion of B cells [30]. Studies have also demonstrated the efficacy of using autologous regulatory B cells in EAE [16,31]. In conclusion, we have not observed a significant deficiency in B cells with a Breg phenotype in MS which Rports the hypothesis that MS does not arise from an acquired Breg numerical defect. Interestingly, we observed that MS subjects, independently of DMD used, have a significant increase in blood B-cell content which Rports the notion that B-cell biology is altered in MS in a manner which may provide rationale for B-cell depletion strategies for this ailment.

## Figures and Tables

**Table 1:** Distribution of the disease types and disease modifying drugs of 32 MS samples.

Drug	Female RMMS	Male RMMS	Female PPMS	Male PPMS	Female SPMS	Male SPMS
**Interferon-beta**	9	2	1	0	0	0
**Glatiramer acetate**	6	1	0	0	0	0
**Fingolimod**	1	1	0	0	0	0
**None**	9	0	0	0	1	1

**RRMS**: relapsing remitting multiple sclerosis, **PPMS**: primary progressive multiple sclerosis, **SPMS**: secondary progressive multiple sclerosis.
